# Advances in prostate-specific membrane antigen-targeted theranostics: from radionuclides to near-infrared fluorescence technology

**DOI:** 10.3389/fimmu.2024.1533532

**Published:** 2025-01-10

**Authors:** Zhongji Jiang, Gaohaer Kadeerhan, Jin Zhang, Wenmin Guo, Hong Guo, Dongwen Wang

**Affiliations:** ^1^ Department of Urology, National Cancer Center/National Clinical Research Center for Cancer/Cancer Hospital Shenzhen Hospital, Chinese Academy of Medical Sciences and Peking Union Medical College, Shenzhen, China; ^2^ School of Medicine, Southern University of Science and Technology, Shenzhen, China; ^3^ Central Laboratory, National Cancer Center/National Clinical Research Center for Cancer/Cancer Hospital & Shenzhen Hospital, Chinese Academy of Medical Sciences and Peking Union Medical College, Shenzhen, China; ^4^ Department of Urology, First Hospital of Shanxi Medical University, Taiyuan, Shanxi, China

**Keywords:** prostate cancer (PCa), prostate-specific membrane antigen (PSMA), near-infrared/shortwave infrared (NIR/SWIR), targeted imaging, fluorescence-guided surgery (FGS), radioligand therapy (RLT), photothermal therapy (PTT), photodynamic therapy (PDT)

## Abstract

Prostate-Specific Membrane Antigen (PSMA) is a highly expressed and structurally unique target specific to prostate cancer (PCa). Diagnostic and therapeutic approaches in nuclear medicine, coupling PSMA ligands with radionuclides, have shown significant clinical success. PSMA-PET/CT effectively identifies tumors and metastatic lymph nodes for imaging purposes, while ^177Lu^-PSMA-617 (Pluvicto) has received FDA approval for treating metastatic castration-resistant PCa (mCRPC). Despite their success, radionuclide-based diagnostic and therapeutic methods face limitations such as high costs and significant side effects. Recently, near-infrared (NIR) fluorescence imaging and phototherapy have advanced significantly in biomedical applications. It’s benefits, such as deep tissue penetration, real-time precision, and minimal side effects, have driven broader clinical adoption, especially in fluorescence-guided surgery (FGS). This review suggests combining NIR dyes with PSMA ligands to enable targeted, high-resolution imaging with superior signal-to-background ratios, facilitating precise FGS. NIR techniques can also aid pathological diagnosis in ex vivo specimens. Furthermore, combining photosensitizers with PSMA ligands allows localized photothermal (PTT) or photodynamic therapy (PDT) under NIR irradiation, producing heat or reactive oxygen species (ROS) to treat PCa. This review aims to extend the clinical success of radionuclide-based PSMA targeting by exploring advances in NIR-based FGS and phototherapy, presenting a promising new diagnostic and therapeutic approach.

## 1 Introduction

Prostate cancer (PCa) is the most prevalent malignancy among men, with American Cancer Society statistics from 2024 identifying it as the second leading cause of cancer-related deaths, significantly burdening healthcare systems and societies worldwide (1). In China, the aging population is a critical factor driving a steady annual rise in PCa incidence (2). Early detection of PCa often involves observing symptoms and detecting elevated prostate-specific antigen (PSA) levels, with fine-needle biopsy regarded as the diagnostic gold standard due to its accuracy (3). Recent technological advancements, such as prostate-specific membrane antigen (PSMA) based PSMA-PET/CT, have been endorsed by the European Association of Urology (EAU) as essential diagnostic tools for PCa, offering superior accuracy in identifying tumors and metastatic lymph nodes (4).

Surgical intervention remains the cornerstone of PCa treatment (5, 6). However, complementary approaches, including androgen deprivation therapy (ADT) and PSMA radioligand therapy (RLT), are gaining prominence for their efficacy in managing advanced cases (7, 8). Simultaneously, advancements in medical technology have transformed surgical methodologies. Traditional open surgeries have evolved into minimally invasive, robot-assisted procedures that achieve real-time cellular and molecular-level precision (7, 9, 10). Despite these advancements, conventional intraoperative reliance on white light to determine resection margins often results in incomplete removal of tumors or overlooked metastatic nodes, leading to the development of fluorescence-guided surgery (FGS). FGS has revolutionized intraoperative imaging, significantly improving precision and outcomes (11). Among emerging imaging techniques, near-infrared (NIR, 700–3000 nm) fluorescence imaging stands out for its ability to provide high-resolution, real-time visualization of molecular targets, advancing both surgical navigation and phototherapy applications (12).

**Figure 1:**
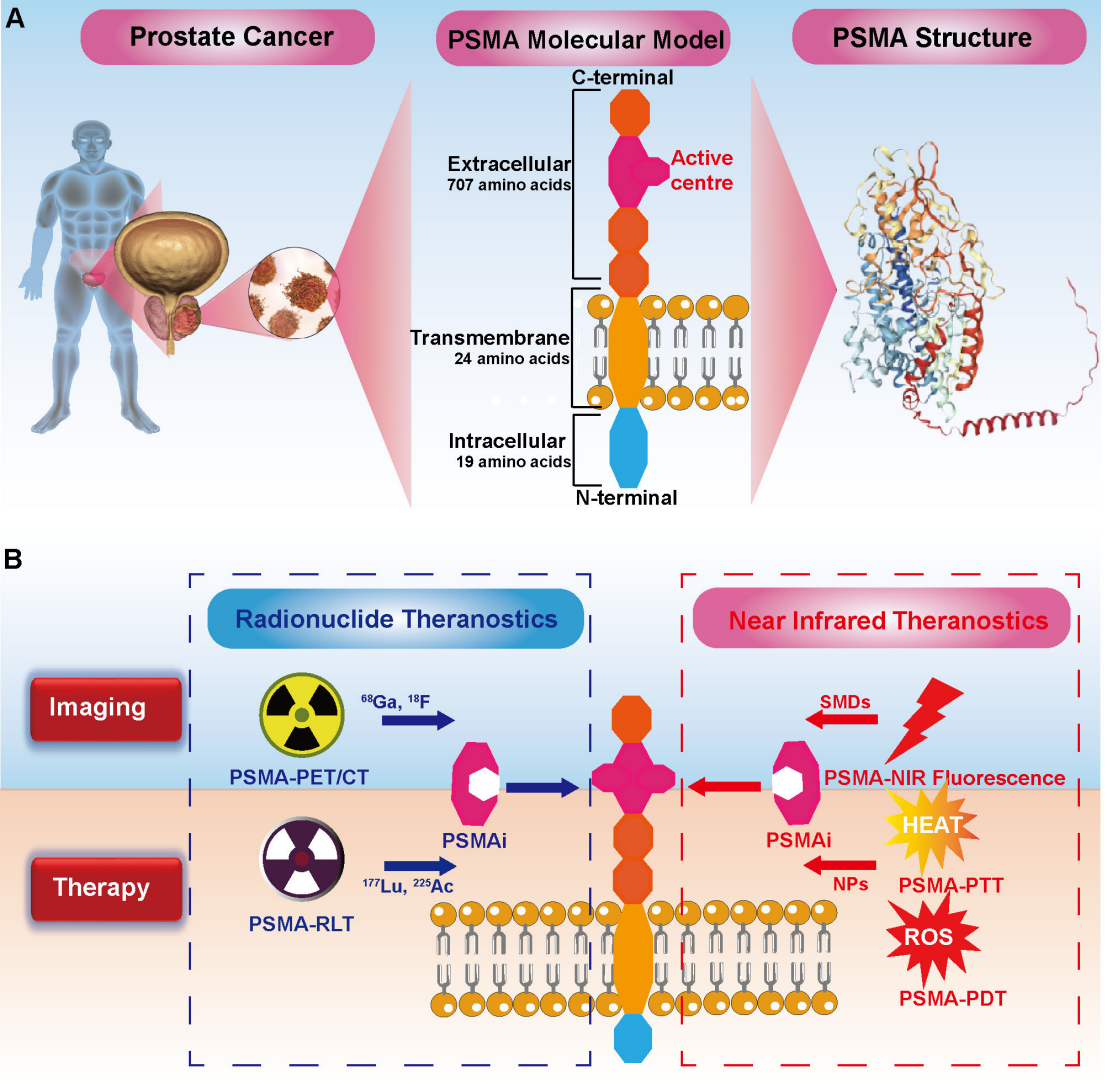
Comprehensive Theranostics of Prostate Cancer (PCa) Targeting PSMA: Bridging Radionuclides and Near-Infrared. **(A)** PSMA structure: The intracellular domain of PSMA consists of 19 amino acids, the transmembrane domain is composed of 24 amino acids, and the extracellular domain contains 707 amino acids. **(B)** Radionuclide Theranostics: PSMA inhibitors (PSMAi) leverage the enzymatic activity of PSMA's extracellular domain to target PCa. It can be labeled with ^68^Ga and ^17^F for imaging, and with ^177^Lu and ^255^Ac for therapy. Notably, ^177^Lu-PSMA-617. Near-Infrared (NIR) Theranostics: NIR dyes, including small molecule dyes (SMDs), are conjugated with PSMAi for PSMA-targeted NIR fluorescence imaging. Photosensitizers in nanoparticles (NPs) linked to PSMAi enable targeted photothermal therapy (PTT) and photodynamic therapy (PDT), generating heat and reactive oxygen species (ROS) to combat cancer cells, respectively.

This mini review emphasizes the critical role of PSMA as a classical PCa target. PSMA has shown exceptional promise in targeted radionuclide imaging and therapy, achieving significant clinical milestones. Moreover, coupling PSMA with NIR imaging not only enhances surgical precision but also introduces innovative applications in pathological diagnosis and targeted phototherapy. These advancements underscore PSMA’s potential in future precision medicine applications, such as FGS and localized photothermal (PTT) therapy or photodynamic therapy (PDT), solidifying its role in the next generation of PCa management strategies (**Figure 1**).

## 2 Structure and biological functions of PSMA

PSMA is a type II transmembrane glycoprotein highly expressed in PCa. It consists of 750 amino acids, organized into three domains: a 19-amino-acid intracellular segment, a 24-amino-acid transmembrane domain, and a 707-amino-acid extracellular domain (13) (**Figure 1A**). PSMA features a distinctive 20 Å-deep funnel-shaped tunnel leading to its active site, which contains two Zn²^+^ ions and substrate-binding pockets named S1 and S1’ (14, 15). This structural uniqueness facilitates the development of targeted ligands, such as monoclonal antibodies, aptamers, and small-molecule inhibitors (16). Functionally, PSMA exhibits enzymatic activities, including carboxypeptidase and folate hydrolase, with its extracellular domain ensuring protection of its active site. In PCa cells, PSMA enhances oncogenic pathways by activating glutamate receptors and the PI3K-AKT pathway, thereby driving tumor progression and metastasis (17).

PSMA is overexpressed in 90% of metastatic PCa cases, with levels up to 1,000 times higher than in benign tissues, correlating strongly with tumor aggressiveness and Gleason scores (18, 19). It is also expressed in the neovasculature of various cancers, such as ovarian and breast cancer (20). These characteristics make PSMA a critical target for fluorescence labeling and therapeutic interventions in PCa. Based on the structure of PSMA, this review examines the advantages and disadvantages of its application in both radionuclide and near-infrared (NIR) technology for the diagnosis and treatment of prostate cancer (**Table 1**).

**Table 1:** Comparison of PSMA-Targeted Radionuclide and Near-Infrared (NIR) Imaging and Therapy.

Characteristics	Radionuclide Imaging and Therapy	NIR Imaging and Therapy
**Imaging Principle**	Utilizes radiation emitted by radionuclides (e.g., gamma rays) for imaging.	Utilizes the interaction between near-infrared light and fluorophores to generate signals.
**Materials**	Includes ^18^F, ^68^Ga, 99Tcm, ^177^Lu, ^225^Ac, etc.	Includes small molecules, gold particles, rare earth nanoparticles, and carbon nanotubes, etc.
**Tissue Penetration**	Limited, with rapid attenuation of high-energy radiation in tissues.	Good, with the NIR-II window enabling deep tissue imaging.
**Real-time Monitoring**	Yes, modalities such as PET/CT allow for real-time monitoring.	Yes, capable of real-time tumor monitoring.
**Specificity**	Good, capable of targeting PSMA.	Good, capable of targeting PSMA.
**Side Effects**	High, due to radiation risks.	Low, with phototherapy being relatively safe.
**Cost and Equipment**	High, requiring expensive nuclear medicine equipment.	Relatively low, but specific fluorescence imaging equipment is needed.
**Therapeutic Applications**	Directly damages tumor cells with emitted radiation.	Photothermal therapy (PTT) and photodynamic therapy (PDT).
**Operational**	Complex, requiring specialized handling of radioactive materials.	Relatively simple, with straightforward fluorescence imaging procedures.
**Clinical Application**	FDA-approved for clinical use.	Undergoing preclinical and clinical trials.

## 3 The current status of PSMA-targeted radionuclide diagnostics and therapy

Radionuclides are integral to medical imaging and radiotherapy, with their application dating back to 1941 when Saul Hertz utilized radioactive Iodine-131 for treating hyperthyroidism. In contemporary oncology, PSMA-targeted radionuclides have revolutionized PCa management (**Figure 1B**). Gallium-68 (^68^Ga) and Lutetium-177 (^177^Lu) labeled PSMA molecules are now routinely employed for diagnosis and treatment, respectively. ^68^Ga-PSMA-PET/CT imaging offers precise tumor and metastasis localization, while ^177^Lu PSMA therapies have demonstrated significant efficacy in extending survival in metastatic PCa patients, as corroborated by numerous clinical studies (21, 22).

### 3.1 PSMA-PET/CT imaging

Over recent decades, the use of PET/CT imaging, which integrates functional and anatomic modalities, has increased in PCa. Targeted PSMA-PET is now widely used in imaging diagnostics for PCa, including early diagnosis, staging, advanced metastasis assessment, and detecting biochemical recurrence post-prostatectomy (23). Several PSMA-based radiotracers, such as ^68^Ga-PSMA-11, ^18^F-PSMA-1007, ^99^Tcm-PSMA I&S, and ^18^F-DCFPyl, have been developed and using in clinical (21–23). A study on biochemically recurrent PCa estimated that PSMA-PET imaging could reduce 75 PCa deaths per 1000 patients, increase life years by 988, and add 824 quality-adjusted life years compared to conventional imaging (4). While PSMA PET/CT with ^68^Ga-PSMA-11 is the most researched and clinically advanced, emerging ^18^F-based methods with lower positron energy and longer half-life may offer higher spatial resolution. Potential future applications include tumor localization, treatment stratification, and efficacy monitoring of advanced PCa. Standardizing PSMA ligand PET imaging interpretation is necessary, and efforts are needed to develop user-friendly, side-effect-free diagnostic methods.

### 3.2 PSMA-radioligand therapy

Some PCa patients, due to health limitations, cannot undergo surgery and instead rely on innovative treatments like PSMA-RLT, which has shown considerable clinical success (22). The ligand ^177^Lu-PSMA-617 binds specifically to PSMA on PCa cells and emits high-energy β and γ particles to kill cancer cells, inducing DNA damage in cancer cells and their surrounding microenvironment. This therapy has been proven to prolong survival and enhance the quality of life in patients with metastatic castration-resistant prostate cancer (mCRPC) (24, 25). PCa exhibits radioresistance, especially in hypoxic conditions. However, α-emitters like ^225^Ac-PSMA-617 release potent α-particles unaffected by hypoxia, offering potential benefits for patients with widespread metastases or prior ^177Lu^-PSMA-617 treatment failures (26). Adverse effects of PSMA-RLT must be closely monitored. ^225^Ac/^177^Lu-PSMA-617 therapy results in significant radioactive uptake in salivary glands, potentially causing glandular damage, dry mouth, and reduced taste function, thereby impacting the quality of life (27, 28). Notably, salivary glands act as dose-limiting organs in PSMA-RLT, constraining higher dose administration and impacting therapeutic outcomes. Furthermore, radiotherapy presents challenges such as high costs, reliance on specialized equipment, and the need for trained personnel. Radionuclides are essential in PCa imaging and treatment but pose significant challenges and side effects as mentioned above. Thus, emerging technologies aim to improve diagnostic and therapeutic outcomes with lower costs and fewer side effects, increasing accessibility for patients.

## 4 Advances in NIR/SWIR applications in the biomedical field

Molecular imaging based on nar-infrared/short-wave infrared (NIR/SWIR) technology is a novel imaging technique that qualitatively and quantitatively investigates physiological processes at the molecular level within living organisms using imaging agents. This technology, characterized by its non-invasive, real-time, dynamic, and quantitative attributes, enables cross-scale visualization monitoring from subcellular to cellular, tissue, organ, and whole organism levels.

### 4.1 NIR-I to NIR-II

Professor Hongjie Dai is a pioneer in Second Near-Infrared Window (NIR-II) imaging, which extends the range from First Near-Infrared Window (NIR-I, 700-900 nm) to NIR-II (1000-3000 nm), further subdivided into NIR-IIa, NIR-IIb, NIR-IIc, and NIR-IId (29, 30). *In vivo* fluorescence imaging in living tissues is affected by absorption, scattering, and autofluorescence. Compared to NIR-I, NIR-II fluorescence imaging minimizes light absorption and scattering, reduces autofluorescence, and enables non-invasive imaging with ultra-high resolution, a high signal-to-background ratio (SBR), and centimeter-level tissue penetration (31). This powerful imaging technique complements clinical modalities like MRI, PET, and X-ray. NIR-II imaging overcomes the limited penetration of other optical methods and excels in molecular imaging. In 2009, Dai’s team introduced carbon nanotubes for NIR-II fluorescence imaging, vividly depicting tumor vasculature and paving the way for advanced NIR-II bioimaging technologies (32). The development of NIR-II probes (1050-1350 nm, 1700 nm) enabled breakthroughs in real-time blood flow monitoring and skull imaging, heralding the shift from NIR-I to NIR-II *in vivo* imaging (33, 34).

### 4.2 Non-specific to specific targeting

Indocyanine green (ICG), a cyanine-based small molecule fluorescent probe approved by the FDA in 1959, is widely used in various fluorescence-guided surgeries (35). ICG has a high molar extinction coefficient, high fluorescence quantum yield, and good biocompatibility, making it useful in intraoperative angiography, lymphangiography, and tumor resection (36). Research, including animal studies, has demonstrated that ICG’s NIR-II fluorescence imaging outperforms NIR-I imaging, facilitating the clinical translation of NIR-II technology (37). However, as a non-targeted probe, ICG cannot differentiate between benign and malignant tumors, and its accumulation in non-target tissues may result in false-positive tumor diagnoses (38). Consequently, there is a need for NIR dyes with specific targeting capabilities.

Several research groups are currently developing NIR probes with specific targeting. Preclinical animal studies and *ex vivo* human tissue experiments have shown promising results (39–43). In 2016, Dai’s team used the rapidly excreted NIR-II fluorophore CH1055 to achieve high SBR imaging of the lymphatic system in mice (44). Additionally, new materials have been developed for tumor molecular imaging in the NIR-II region, targeting PD-L1, high endothelial venules (aMECA-79), CD169+ macrophages, and CD3+ T cells in lymph nodes (45–47). These advancements enable non-invasive molecular imaging at the single-cell or vessel level and have also facilitated *in vivo* imaging of cancer vaccines and immune responses (31). This progress marks a shift from non-specific to specific targeting, offering promising potential for tumor-specific FGS.

### 4.3 Basic research to clinical application

In NIR FGS, small molecules, gold particles, rare earth nanoparticles, and carbon nanotubes are being extensively studied as NIR-II fluorescent dyes in clinical and preclinical trials (29, 48). Studies using CD105-targeted molecular imaging probes have achieved a tumor-to-muscle ratio of up to 300, allowing precise tumor visualization and removal of residual cancer cells, ensuring thorough yet conservative resections and setting a benchmark for imaging-guided surgery (49). The gold particle-phosphatidylcholine (Au-PC) probe minimizes interactions with serum proteins, cells, and tissues, enabling accurate lymph node localization. This novel fluorescent technology shows strong potential for clinical application (50). NIR-II molecular imaging excels in deep tissue imaging and shows high clinical translation potential. Advances include new fluorescent probes, preclinical disease models (e.g., cancer, cardiovascular, neural), and clinical applications like perfusion imaging, lymph node localization, immunotherapy, photodynamic therapy, and FGS, paving the way for a novel surgical paradigm. Despite significant progress, most tumor-targeting NIR fluorescent probes remain in preclinical stages. Notably, in 2021, Pafolacianine (OTL38) became the first FDA-approved tumor-targeting fluorescent imaging agent for clinical use, marking a milestone in the field (51).

## 5 Targeted PSMA NIR imaging applications: fluorescence-guided surgery and ex vivo tissue assessment

Early screening is essential for diagnosing and treating PCa, as early PSA screening significantly reduces the mortality risk in these patients. Recently, PSMA-PET/CT has been endorsed by the EAU, the European Society for Medical Oncology (ESMO), and the Chinese Urological Association as a vital method for early diagnosis and for evaluating PCa recurrence and metastasis (52). Ultrasound-guided fine-needle aspiration biopsy remains the gold standard for diagnosing PCa.

### 5.1 Fluorescence-guided surgery

**Figure 2:**
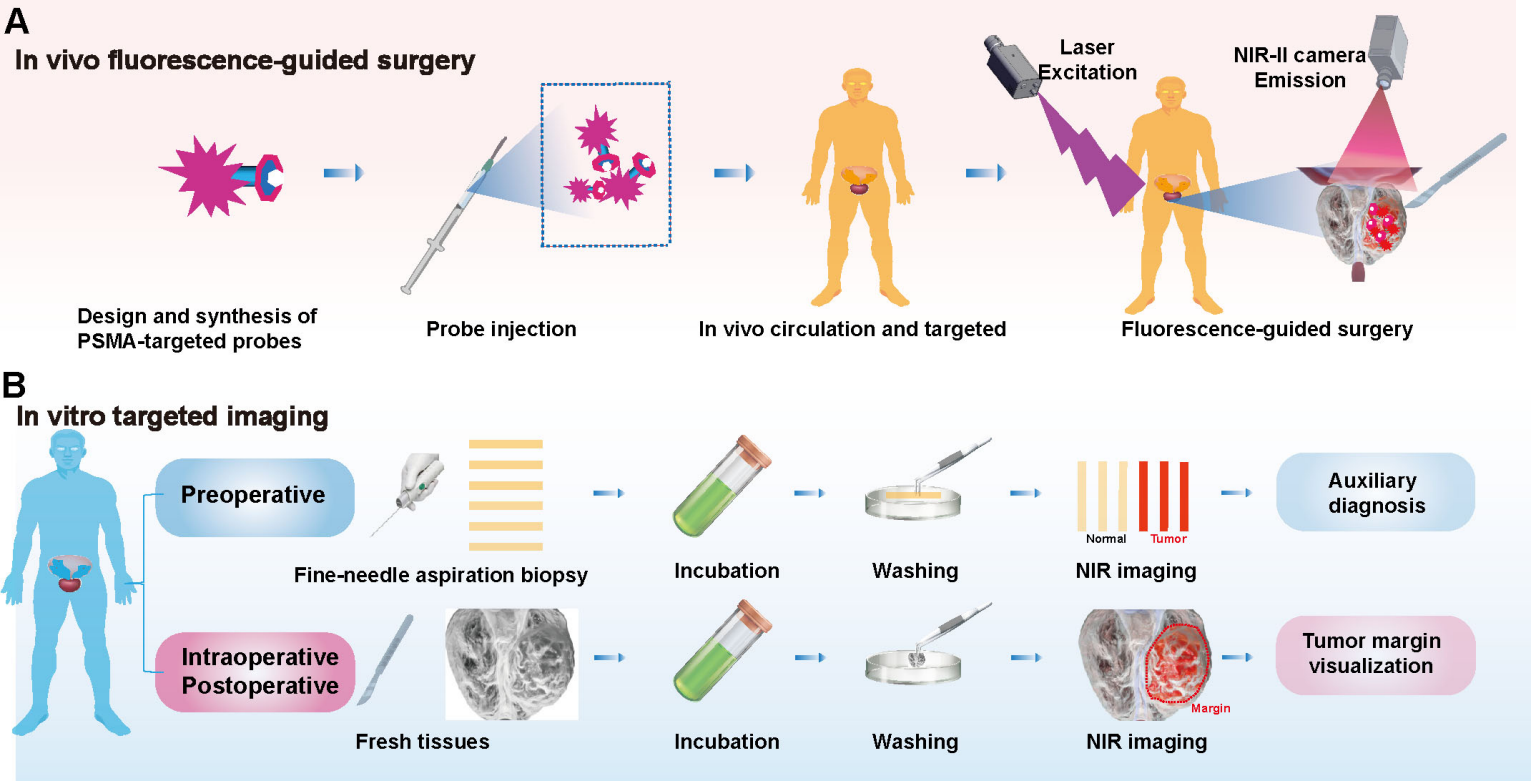
Targeted PSMA NIR Applications: Fluorescence-guided surgery and *in vitro* tissue assessment. **(A)** *In Vivo* Fluorescence-Guided Surgery: PSMA-specific fluorescent probes are designed for NIR imaging. Post-injection, these probes target tumor sites and are visualized using a NIR laser, particularly in the NIR-II region. **(B)** *In vitro* targeted imaging: In the context of PCa, needle biopsy samples from preoperative patients are incubated with PSMA-targeted probes, washed, and imaged. Additionally, this technique can be applied to intraoperative and postoperative specimens to define tumor margins, informing surgical and prognostic assessments.

Following diagnosis, PCa patients frequently undergo radical prostatectomy, with some requiring extended pelvic lymph node dissection (ePLND). Gandaglia et al. reported that combining PSMA-radioguided surgery (PSMA-RGS) with ePLND provides high sensitivity and specificity for identifying lymph nodes during surgery (53). However, the radiation exposure from this surgical method may pose risks to both surgeons and patients, highlighting NIR imaging as a safer and more promising alternative. In NIR imaging, researchers initially developed PSMA-specific fluorescent probes capable of circulating in the body and targeting tumor sites (54, 55). Signals collected under NIR laser (especially in the NIR-II region) enable deep, high-resolution imaging with a high SBR for precise fluorescence-guided tumor resection (**Figure 2A**). For example, the PSMA-targeting fluorescent probe OTL78 was developed by conjugating the high-affinity PSMA ligand DUPA with the commercial NIR dye S0456. This probe achieved a tumor-background ratio (TBR) of 5:1 *in vivo*, effectively detecting small tumors (56). Despite its good biosafety, the phase II clinical trial also found that OTL78 had low sensitivity for detecting tumor-positive margins (48.6%) and limited ability to identify positive lymph nodes (64.3%) during surgery (57–59). Another NIR probe, Cy-KUE-OA, specific to PSMA and cell membrane phospholipids, was developed and validated in ex vivo imaging studies, showing potential for clinical application in FGS for prostate cancer (60). Current challenges such as probe size, extended circulation times, and the limitation to the NIR-I region (54, 55) underscore an urgent need for the development and clinical application of NIR-II probes specific for PCa in urological surgery.

### 5.2 *In vitro* tissue rapid visualization

Determining tumor boundaries during surgery remains challenging. Rapid pathological testing provides a diagnosis within 30 minutes but is limited by the morphological similarity of tumor cells in HE staining, leading to potential misdiagnoses and complications. Immunohistochemical staining (IHC) supports diagnosis but requires 2–3 hours, limiting intraoperative utility and risking missed small lesions (61). NIR-specific probes have shown promise in preclinical and clinical studies for visualizing tumor boundaries in ex vivo tissues, including lung, osteosarcoma, and colorectal cancers (41, 43, 62). In PCa, incubating preoperative needle biopsy strips with probes enables visualization of tumor tissues while sparing normal tissues, offering a potential diagnostic tool. Imaging intraoperative specimens can delineate tumor boundaries, aiding surgical and prognostic assessments (**Figure 2B**). Probes’ incubation times range from minutes to hours, with shorter times being clinically advantageous. PSMA-N064 solution and Cy-KUE-OA have demonstrated effective tumor visualization in PCa but require further development to match rapid pathological testing (60, 63). NIR-I imaging devices have achieved clinical maturity and are widely utilized. In contrast, NIR-II imaging systems and dyes offer the potential for enhanced image clarity. The adoption of NIR-II technology in clinical settings could herald a revolutionary advance in clinical imaging (48, 64).

## 6 Precision Phototherapy Targeted PSMA: photothermal therapy and photodynamic therapy

Phototherapy has recently gained considerable attention for its non-invasive nature, precise spatiotemporal control, minimal side effects, and low resistance. Combining PDT with PTT allows their complementary mechanisms to synergistically enhance therapeutic effects (65). Targeted strategies enhance the delivery of photothermal and photodynamic agents to tumors, increasing the selectivity and effectiveness of PTT and PDT (66). Passive targeting involves modifying nanoparticle or macromolecule size and surface chemistry to leverage the Enhanced Permeability and Retention (EPR) effect. Active targeting employs high-affinity ligands to bind specific molecules overexpressed on cancer or tumor epithelial cells, such as PSMA, EGFR, and B3-H7.

### 6.1 Photothermal therapy

Photothermal therapy (PTT) is an advanced technique that uses NIR light of specific wavelengths to activate photosensitive nanomaterials, converting light energy into heat to kill tumor cells or induce apoptosis, thus inhibiting tumor growth. PTT allows precise temperature control by adjusting laser power. The temperature increase to 41–43°C induces hyperthermia, which does not directly kill tumors but enhances the effects of radiotherapy and chemotherapy. Higher temperatures, including subcoagulation (43–55°C) and coagulation (55–100°C), cause rapid cell death by protein denaturation and cell membrane damage (67). NIR light in PTT penetrates tissues deeply while minimizing damage to normal tissues. Compared to traditional methods, PTT is minimally invasive, efficient, precise, and effective in inhibiting tumor metastasis (68). Pathological and biochemical recurrences frequently occur after PCa surgery. Combining mitochondrial-targeted NIR-II FGS with intraoperative PTT significantly reduces recurrence rates (69). PSMA-targeted PTT probes hold significant potential for further development, leveraging imaging for therapeutic applications.

### 6.2 Photodynamic therapy

Photodynamic therapy (PDT) is an important phototherapy method. Upon absorbing incident light, photosensitizer (PS) molecules transition from the ground state to a short-lived excited singlet state (~ns), followed by a more stable excited triplet state (~ms). From the triplet state, PS molecules can return to the ground state via Type I or Type II photodynamic reactions. Type I reactions involve electron transfer from activated PS to substrates, generating reactive oxygen species (ROS). In Type II reactions, energy is transferred directly to ground-state molecular oxygen, producing highly reactive singlet oxygen (^1^O_2_). These reactive species trigger various downstream biological events, such as cytotoxicity, inflammation, and vascular damage. Due to the limited diffusion range of ROS, targeted PDT can provide precise treatment for solid tumors (65). Researchers have synthesized PSMA-N064, a targeted photosensitizer IRDye700DX, which generates reactive oxygen under laser irradiation, inducing tumor cell apoptosis, delaying growth, and improving survival in PSMA-positive tumor mice (63). In fresh ex vivo human PCa tissue, PSMA-PDT treatment significantly increased cell apoptosis compared to untreated controls (63). PDT is limited by the hypoxic nature of solid tumors, while PTT carries the risk of off-target damage. The combination of PDT and PTT, whether applied simultaneously or sequentially, optimizes the benefits of both approaches while mitigating their individual limitations. PCa, traditionally considered a “cold tumor,” may be converted into a “hot tumor” through activation by PTT or PDT. Additionally, PSMA-targeted NIR phototherapy can be integrated with ADT and chemoradiotherapy to improve therapeutic outcomes.

## 7 Conclusion and future perspectives

PSMA is a key target for diagnosing and treating PCa and has been widely used in nuclear medicine, showing great promise for NIR-based imaging and phototherapy. We discuss the diverse characteristics of PSMA-targeted radionuclide and NIR imaging and therapy, as outlined in **Table 1**. PSMA-targeted NIR technology shows promise as a novel clinical approach, offering precise targeting, deep tissue penetration, and minimal side effects, and is anticipated to provide more precise and effective treatment options for patients with PCa.
